# *Cornus officinalis* Seed Extract Inhibits AIM2-Inflammasome Activation and Attenuates Imiquimod-Induced Psoriasis-like Skin Inflammation

**DOI:** 10.3390/ijms24065653

**Published:** 2023-03-15

**Authors:** Se-Bin Lee, Ju-Hui Kang, Eun-Jung Sim, Ye-Rin Jung, Jeong-Hyeon Kim, Prima F. Hillman, Sang-Jip Nam, Tae-Bong Kang

**Affiliations:** 1 BK21 Project Team, Department of Applied Life Science, Graduate School, Konkuk University, Chungju 27478, Republic of Korea; 2 Department of Chemistry and Nanoscience, Ewha Womans University, Seoul 03760, Republic of Korea; 3 Department of Biotechnology, Research Institute of Inflammatory Diseases, Research Institute (RIBHS), College of Biomedical and Health Science, Konkuk University, Chungju 27478, Republic of Korea

**Keywords:** AIM2 inflammasome, *Cornus officinalis*, macrophage, IMQ-induced psoriasis-like skin inflammation

## Abstract

The AIM2 inflammasome is an innate immune system component that defends against cytosolic bacteria and DNA viruses, but its aberrant activation can lead to the progression of various inflammatory diseases, including psoriasis. However, there have been few reports of specific inhibitors of AIM2 inflammasome activation. In this study, we aimed to investigate the inhibitory activity of ethanolic extracts of seeds of *Cornus officinalis* (CO), a herb and food plant used in traditional medicine, on AIM2-inflammasome activation. We found that CO inhibited the release of IL-1β induced by dsDNA in both BMDMs and HaCaT cells, but that it showed no effect on the release of IL-1β induced by NLRP3 inflammasome triggers, such as nigericin and silica, or the NLRC4 inflammasome trigger flagellin. Furthermore, we demonstrated that CO inhibited the cleavage of caspase-1, an inflammasome activation marker, and an upstream event, the translocation and speck formation of ASC. In addition, further experiments and mechanistic investigations revealed that CO can inhibit AIM2 speck formation induced by dsDNA in AIM2-overexpressing HEK293T cells. To verify the correlation in vivo, we investigated the efficacy of CO in an imiquimod (IMQ)-induced psoriasis model, which has reported associations with the AIM2 inflammasome. We found that topical application of CO alleviated psoriasis-like symptoms, such as erythema, scaling, and epidermal thickening, in a dose-dependent manner. Moreover, CO also significantly decreased IMQ-induced expression of AIM2 inflammasome components, including AIM2, ASC, and caspase-1, and led to the elevation of serum IL-17A. In conclusion, our results suggest that CO may be a valuable candidate for the discovery of AIM2 inhibitors and the regulation of AIM2-related diseases.

## 1. Introduction

Inflammasomes are multiprotein complexes in cytosol that defend against various pathogens [1]. The complexes are composed of sensors that detect pathogens, an inflammatory enzyme called capase-1, and, in some cases, an adaptor molecule called ASC [2,3]. Several sensors have been identified, including members of the pyrin domain-containing NLR family (NLRP1 and NLRP3), absent in melanoma 2 (AIM2), the NLR family apoptosis inhibitory protein (NAIP)/NLR family, and caspase activation and recruitment domain (CARD) containing 4 (NLRC4), which have been widely studied [4]. When triggers from pathogens bind to these sensors, protein complexes are formed, which activate caspase-1 through autocleavage [5,6]. The activated caspase-1 cleaves pro-inflammatory cytokines, such as pro-IL-1β and pro-IL-18, converting them into their bioactive forms, which induce inflammation [7].

However, inflammasome activation can occur in the absence of infection, leading to unnecessary and prolonged inflammatory responses that may contribute to the progression of various inflammatory diseases [8,9]. Therefore, there have been many efforts to identify compounds that can regulate inflammasome activation from various sources, such as chemical libraries and medicinal plants, and these efforts have been particularly focused on NLRP3 inhibitors, which are known to have significant associations with several metabolic diseases, such as gout, diabetes, and cardiovascular disease [10,11,12].

AIM2 is a cytosolic sensor that activates inflammasomes upon recognizing dsDNA derived from viruses and bacteria [13,14,15,16]. However, it also responds to dsDNA from damaged self-cells and tissues, leading to unnecessary inflammatory responses that contribute to the progression of diseases, such as psoriasis, cancer, and atherosclerosis [17,18,19,20,21,22].

Therefore, this study investigated the inhibitory effects of plant extracts that have been used in traditional medicine on AIM2 inflammasomes and identified that the extract of *Cornus officinalis* seed has an inhibitory effect on AIM2 inflammasomes. *Cornus officinalis* is a flowering plant species of the dogwood family that is used in traditional medicine and as a food source in East Asia [23]. Previous studies have reported various biological activities of *Cornus officinalis*, such as anti-inflammatory, antioxidant, and neuroprotective effects, but its potential as an inflammasome inhibitor has not yet been explored [24,25,26,27,28]. We examined the inhibitory effects of *Cornus officinalis* seed extracts on AIM2 inflammasomes and its mechanisms of action within cells, as well as its in vivo efficacy in an imiquimod-induced psoriasis model.

## 2. Results

### 2.1. Identification of Compounds of CO in LC-MS Fingerprinting

**Figure 1:**
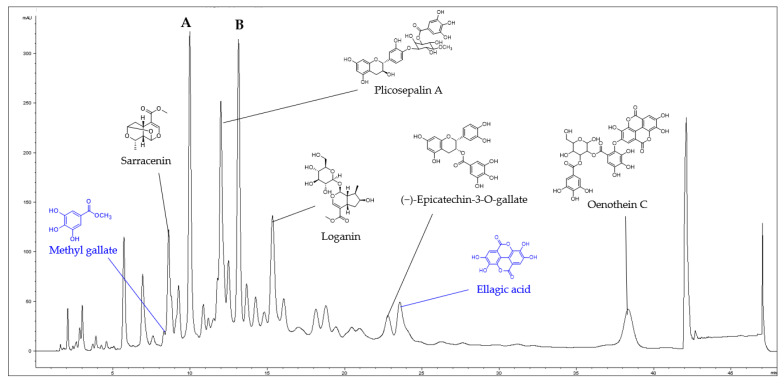
LC-MS fingerprint of the CO (detected at 254 nm). Methyl gallate (*t_R_* = 8.3 min), sarracenin (*t_R_* = 8.6 min), plicosepalin A (*t_R_* = 12.0 min), loganin (*t_R_* = 15.1 min), (−)-epicatechin-3-*O*-gallate (*t_R_* = 22.8 min), ellagic acid (*t_R_* = 23.5 min), and oenothein C (*t_R_* = 38.4 min), A and B (not identified).

The presence of seven compounds in CO were determined from the interpretation of LC-MS and NMR spectroscopic data. Sarracenin, plicosepalin A, loganin, (−)-epicatechin-3-*O*-gallate, and oenothein C were identified from the comparison of the mass data of five compounds with previously reported ones (Figure S1) [29,30]. Meanwhile, HPLC-UV-guided isolation of CO yielded a compound, and the chemical structure was identified as ellagic acid by comparing NMR, MS, and UV spectral data (Figures S2–S4). In addition, methyl gallate was identified from the comparison of retention time and mass data with the standard (Figure S5). However, the major peaks (A and B) shown in the LC-MS were not able to be identified because they easily decomposed during the isolation (Figure 1).

### 2.2. CO Inhibits the Release of IL-1β and Cell Death of Macrophages Induced by Intracellular Poly(dA:dT)

**Figure 2:**
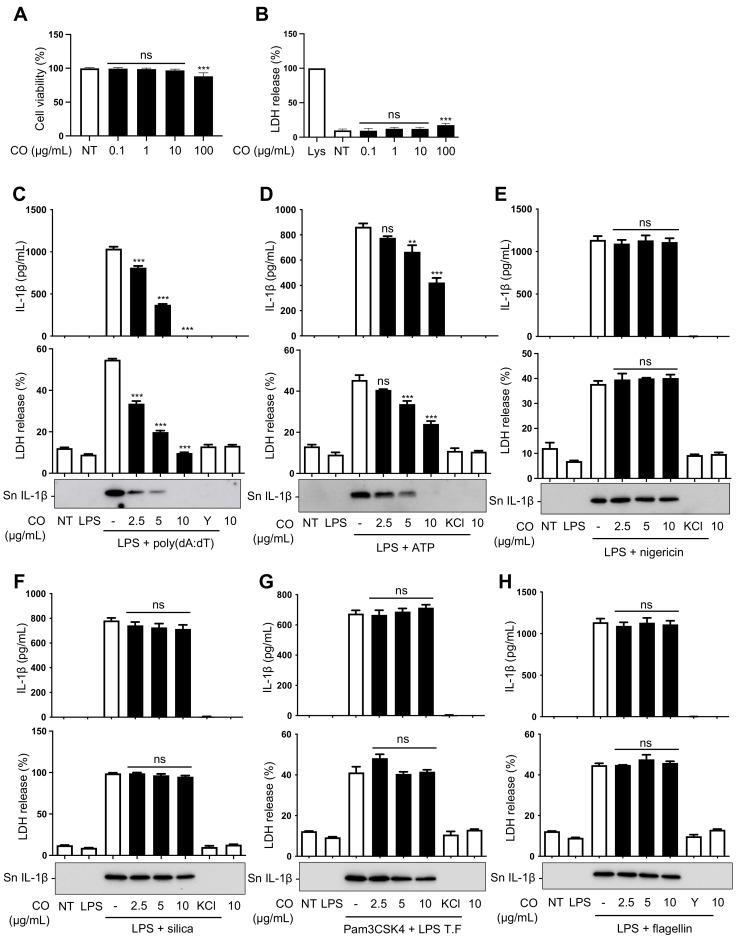
CO inhibits the release of IL-1β and the cell death of LPS-primed BMDMs induced by intracellular poly(dA:dT). (**A**,**B**) Cell viability of BMDMs treated with the indicated concentrations of CO and measured by MTT and LDH assays. Data represent the means ± SDs of three independent experiments performed in triplicate; ** *p* < 0.01, *** *p* < 0.001, ns (non-significant) compared with the non-treated group. (**C**–**F**,**H**) LPS (100 ng/mL)-primed BMDMs were pre-treated with CO or 50 μM YVAD (Y) or KCl (150 mM) for 30 min and treated with (**C**) poly(dA:dT) transfection (1 μg/mL) for 2 h, (**D**) ATP (5 mM) for 1 h, (**E**) nigericin (10 μM) for 1 h, (**F**) silica (150 μg/mL) for 3 h, and (**H**) flagellin (1 μg/mL) transfection for 3 h. (**G**) Pam3CSK4 (300 ng/mL)-primed BMDMs were pre-treated with CO or KCl, as described above, then transfected with LPS (50 μg/mL) for 3 h. (**C**–**H**) Levels of IL-1β in the culture supernatants were assessed by immunoblot or ELISA assay. Occurrence of pyroptotic cell death was assessed by LDH assay. Data represent the means ± SEMs of three independent experiments performed in triplicate; ** *p* < 0.01, *** *p* < 0.001, ns (non-significant) compared with the LPS-primed BMDMs treated with the respective triggers.

A screening study of 110 plant extracts, which are traditionally known to show anti-inflammatory activities, found that the seed extract of *Cornus officinalis* (CO) has an inhibitory effect on AIM2-inflammasome activation. Therefore, follow-up studies were carried out to verify its activity. The non-cytotoxic concentration of CO was determined through MTT and LDH assays performed on mouse bone-marrow-derived macrophages (BMDMs). The results show that CO is not toxic up to 10 μg/mL (Figure 2A,B); thus, this concentration was used for subsequent experiments.

Since the release of cleaved IL-1β into culture supernatants and pyroptotic cell death are hallmarks of inflammasome activation [31], we assessed whether CO could inhibit IL-1β release and cell death of BMDMs stimulated with various stimuli. The results showed that CO inhibited the release of IL-1β and cell death in a dose-dependent manner in response to poly(dA:dT) stimulation but that it had a mild inhibitory effect on ATP-induced IL-1β release and cell death (Figure 2C,D). However, CO had no effect on inflammasome activation triggered by nigericin, silica, LPS transfection, or flagellin (Figure 2E–H).

These results indicate that CO preferentially inhibits AIM2 inflammasome activation triggered by poly(dA:dT) and that there was a mild or lack of effect on inflammasome activation of other types of inflammasome, such as NLRP3 and NLRC4 stimuli.

### 2.3. CO Suppresses the Release of Cleaved IL-1β through the Inhibition of Caspase-1 Activation

**Figure 3:**
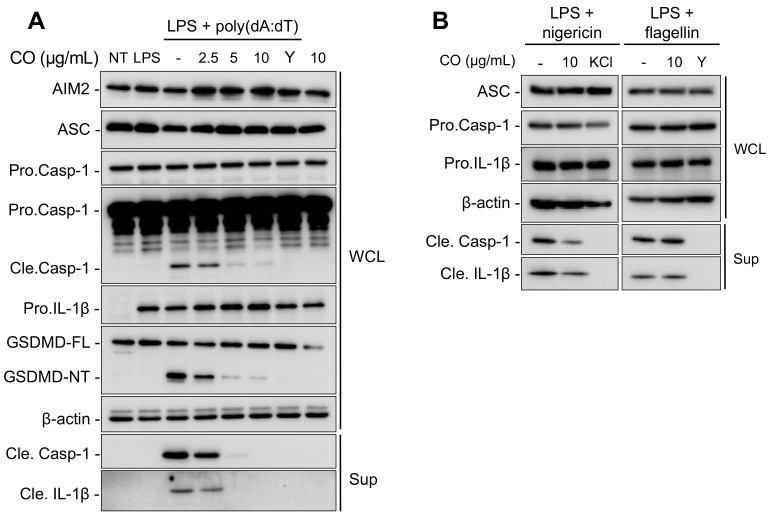
CO suppresses the release of IL-1β through the inhibition of caspase-1 activation. Immunoblotting analysis of culture supernatants (Sup) and whole cell lysates (WCL), with results shown for LPS (100 ng/mL)-primed BMDMs pre-treated with CO or 50 μM YVAD (Y) at the indicated concentrations for 30 min followed by (**A**) poly(dA:dT) (1 μg/mL) transfection for 2 h, (**B**) nigericin (10 μM) treatment for 1 h, and flagellin (1 μg/mL) transfection for 3 h.

The transcriptional upregulation of inflammasome components and the activation of pro-caspase-1 are considered upstream signals that lead to the maturation of pro-IL-1β in the inflammasome activation pathway [32]. To investigate the effect of CO on the expression of inflammasome components and on caspase-1 activation, we examined their levels in LPS-primed mouse bone-marrow-derived macrophages (BMDMs) upon stimulation with poly(dA:dT), nigericin, or flagellin in the presence or absence of CO. Western blotting was used to analyze the expression of AIM2, NLRP3, and NLRC4 inflammasome components. The results show that CO had no effect on the expression of these components (Figure 3A,B). Nevertheless, CO treatment significantly decreased the activation of capase-1 and the cleavage of its substrates, pro-IL-1β and gasdermin-D (GSDMD), in poly(dA:dT)-treated BMDMs (Figure 3A).

These findings suggest that inhibitory effects of CO on AIM2 inflammasome activation are not due to changes in the transcript-expression levels of inflammasome components but, rather, the inhibition of caspase-1 activation.

### 2.4. CO Inhibits AIM2 Inflammasome Activation via the Suppression of ASC Speck Formation

**Figure 4:**
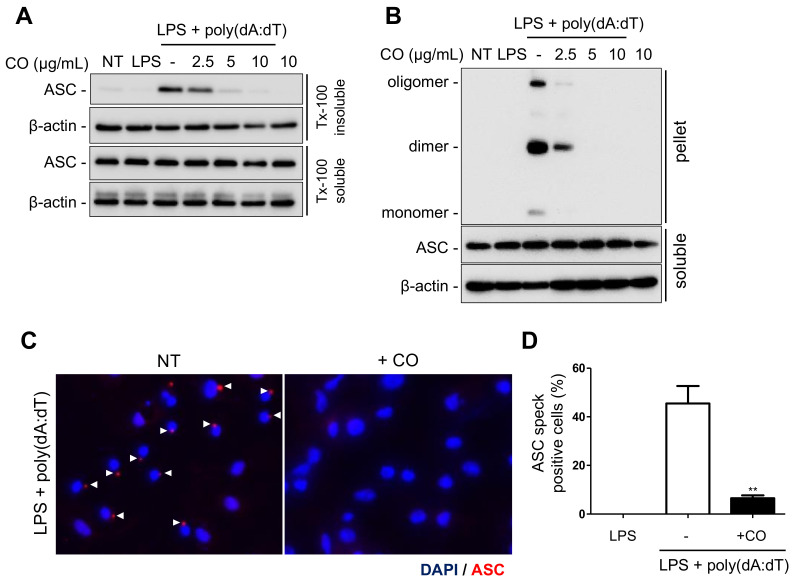
CO inhibits ASC speck formation during AIM2 inflammasome activation. (**A**–**D**) LPS (100 ng/mL)-primed BMDMs were pre-treated with CO for 30 min, then transfected with poly(dA:dT) (1 μg/mL) for 2 h. Western blots of (**A**) ASC translocation from Triton X-100 soluble fraction to insoluble fraction, (**B**) ASC oligomerization in the insoluble fraction (pellet), and (**C**) ASC speck images at 400× magnification upon stimulation with poly(dA:dT). ASC and nuclei are depicted in red and blue (DAPI), respectively. White arrowheads point to ASC speck. (**D**) Quantification of cells with ASC specks showed the percentage of positive cells. At least three fields and more than 100 cells were counted for each condition. Data represent the means ± SDs of three independent experiments; ** *p* < 0.01.

To elucidate the mechanism by which CO inhibits AIM2 inflammasome activation, we examined the response of key steps in the AIM2 inflammasome signaling pathway. The adaptor protein ASC plays a critical role in inflammasome activation by physically linking sensors and caspase-1 [33]. Upon activation, ASC translocates into a Triton X-100 insoluble fraction, where it undergoes oligomerization and forms a multiprotein complex known as ASC speck [16,34,35]. Therefore, we investigated whether CO could inhibit these ASC changes in LPS-primed mouse bone-marrow-derived macrophages (BMDMs) treated with poly(dA:dT) in the presence or absence of CO. The results show that CO inhibited ASC translocation in a dose-dependent manner (Figure 4A). Furthermore, CO suppressed the ASC oligomerization and speck formation induced by poly(dA:dT) (Figure 4B–D).

These findings suggest that CO inhibits AIM2 inflammasome activation by targeting the upstream events of ASC translocation and oligomerization, which subsequently results in the inhibition of caspase-1 activation.

### 2.5. CO Inhibits AIM2 Speck Formation

The binding of AIM2 with dsDNA induces AIM2 oligomerization and speck formation, which is crucial for AIM2 inflammasome complex formation [33,36].

**Figure 5:**
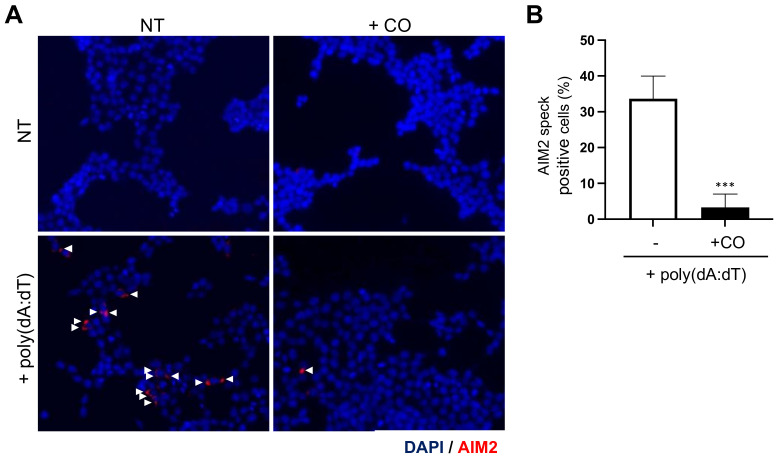
CO inhibits AIM2 speck formation resulting from poly(dA:dT) stimulation. (**A**,**B**) HEK293T cells were transfected with a FLAG-tagged AIM2 expression vector 24 h prior to the experiment. Cells were pre-treated with CO (10 μg/mL) for 30 min, then stimulated with poly(dA:dT) (1 μg/mL) for 4 h. (**A**) AIM2 speck images at 400× magnification upon stimulation with poly(dA:dT) in cells expressing FLAG-tagged AIM2. AIM2 and nuclei are depicted in red and blue (DAPI), respectively. White arrowheads point to AIM2 speck. (**B**) Statistical analysis of the AIM2 specks showing the percentage of positive cells. At least three fields and more than 100 cells were counted for each condition. Data represent the means ± SDs of three independent experiments; *** *p* < 0.001.

To investigate the effect of CO on AIM2 speck formation, AIM2-overexpressing HEK293T cells were treated with poly(dA:dT) to induce AIM2 speck formation in the presence or absence of CO. The cells were then analyzed by immunocytochemistry, and the results showed that CO significantly decreased poly(dA:dT)-induced AIM2 speck formation (Figure 5A,B).

These data suggest that CO inhibits AIM2 inflammasome activation through the inhibition of AIM2 speck formation.

### 2.6. CO Inhibits AIM2-Mediated IL-1β Release from HaCaT Cells and Attenuates IMQ-Induced Psoriasis-Like Skin Pathology

**Figure 6:**
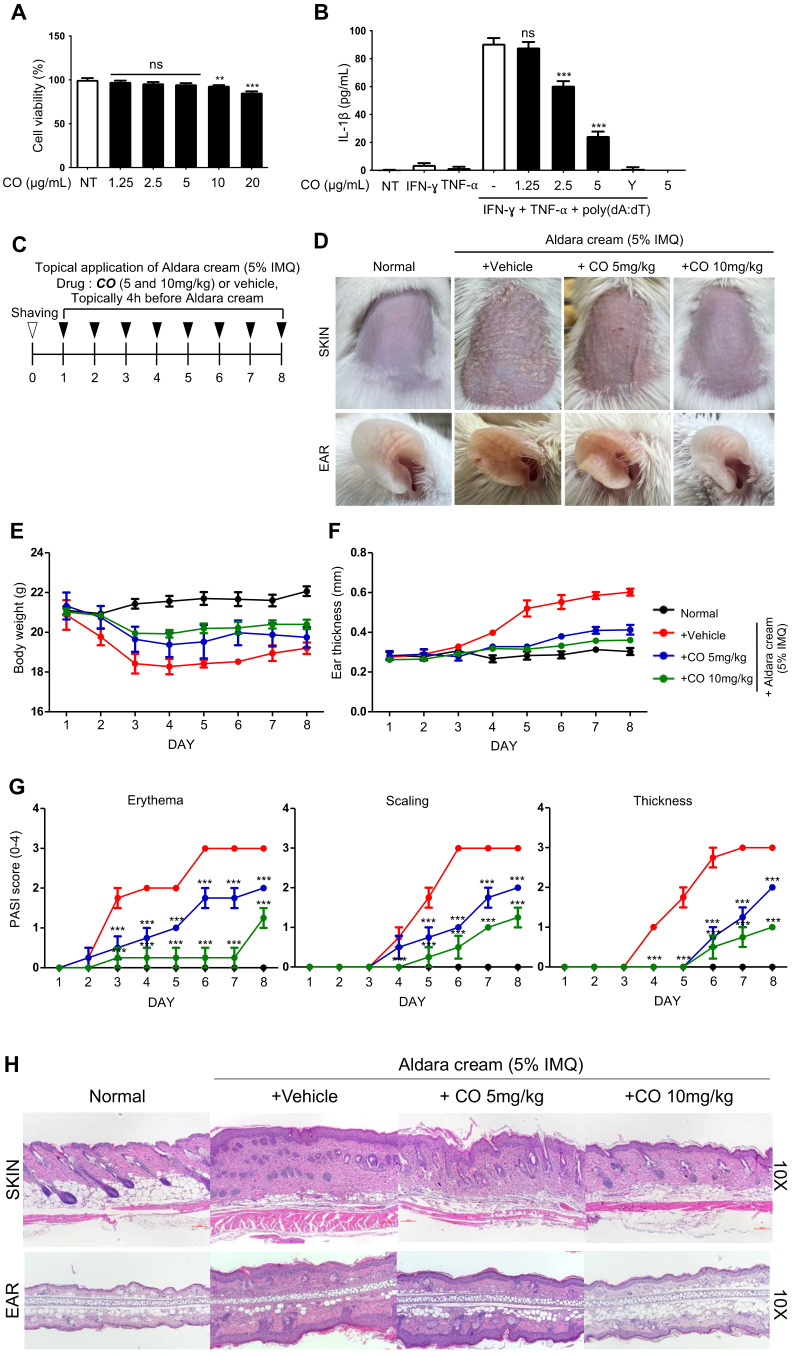
CO inhibits IMQ-induced skin pathology. (**A**) Cell viability of HaCaTs treated with the indicated concentrations of CO for 24 h measured by MTT assay. Data represent the means ± SDs of three independent experiments performed in triplicate; ** *p* < 0.01, *** *p* < 0.001, ns (non-significant) compared with the non-treated group. (**B**) IL-1β concentrations in the culture supernatants of HaCaTs were primed with IFN-γ (100 ng/mL) and TNF-α (10 units/mL) and pre-treated with CO or 50 μM YVAD (Y) at the indicated concentrations for 30 min, followed by transfection with poly(dA:dT) (1 μg/mL) for 24 h. IL-1β concentrations measured by ELISA assay. Data represent the means ± SEMs of three independent experiments performed in triplicate; *** *p* < 0.001, ns (not significant) compared with the IFN-γ and TNF-α-primed HaCaTs treated with the respective poly(dA:dT). (**C**) Schematic presentation of preparing the model for IMQ-induced psoriasis. BALB/c mice received topical applications of CO (5 or 10 mg/kg) or vehicle for 8 consecutive days. Each mouse had Aldara cream (5% IMQ) simultaneously applied on its shaved back skin and right ear for 8 consecutive days to induce psoriasis. (**D**) Phenotypic presentation of a mouse’s back skin and ear after 8 days of treatment, with data for consecutive days on (**E**) body weight, (**F**) ear thickness of mice, and (**G**) erythema, scaling, and thickness scores (PASI) for the shaved back skin. Data represent the means ± SDs; ** *p* < 0.01, *** *p* < 0.001, compared with the vehicle group. (**H**) H&E staining of skin and ear sections in BALB/c mice after 8 days of topical treatment with CO and Aldara cream.

To determine whether CO also affects inflammasome activation in other cell types beyond BMDMs, its activity was tested in human keratinocyte cells (HaCaTs), which are known to express AIM2 and release IL-1β upon stimulation with double-stranded DNA (dsDNA) [37]. To ensure that it did not impact cell viability (Figure 6A), a concentration of less than 10 μg/mL of CO was applied in the subsequent experiments with HaCaT cells.

HaCaT cells were first treated with IFN-γ and TNF-α, then stimulated with poly(dA:dT) transfection to induce AIM2 inflammasome activation [37,38]. In accordance with the results observed in BMDMs, CO also inhibited the poly(dA:dT)-induced secretion of IL-1β in HaCaT cells (Figure 6B).

Prior studies showed that excessive cytosolic DNA can trigger AIM2 inflammasome activation in keratinocytes in psoriatic lesions and contribute to the progression of psoriasis [21,38]. Therefore, to investigate whether CO treatment could reduce psoriatic-like skin inflammation, mice were pre-treated with CO or a vehicle and then exposed to Aldara cream containing 5% imiquimod for 8 consecutive days to induce psoriasis (Figure 6C) [39].

Mice treated with the vehicle showed typical signs of skin inflammation, including erythema, scaling, and thickening of the back skin and ears (Figure 6D–G). However, CO treatment reduced the severity of the skin lesions in a dose-dependent manner and also reduced body weight loss and ear swelling caused by the Aldara cream (Figure 6D–F). The psoriasis area and severity index (PASI) scores showed that CO significantly reduced the erythema, scaling, and thickness (Figure 6G). Finally, the histological analysis showed that CO treatment also reduced parakeratosis, hyperpigmentation, and hair follicle destruction caused by the Aldara cream (Figure 6H).

Together, these results indicate that CO has a protective effect against imiquimod-induced psoriasis.

### 2.7. CO Reduces the Levels of IL-17A and AIM2 Inflammasome Components in IMQ-Induced Skin Lesions

During IMQ-induced skin inflammation, the expression of AIM2 inflammasome components, such as AIM2, ASC, and pro-caspase-1, in skin lesions is upregulated [21,40], and serum levels of IL-17A, a cytokine which has been implicated in the pathogenesis of psoriasis, are increased [41,42,43,44,45]. In order to gain further insight into how CO ameliorates IMQ-induced skin inflammation, we examined the impact of CO on the expression of AIM2 inflammasome components in skin lesions and of serum IL-17A in the mice treated with IMQ.

**Figure 7:**
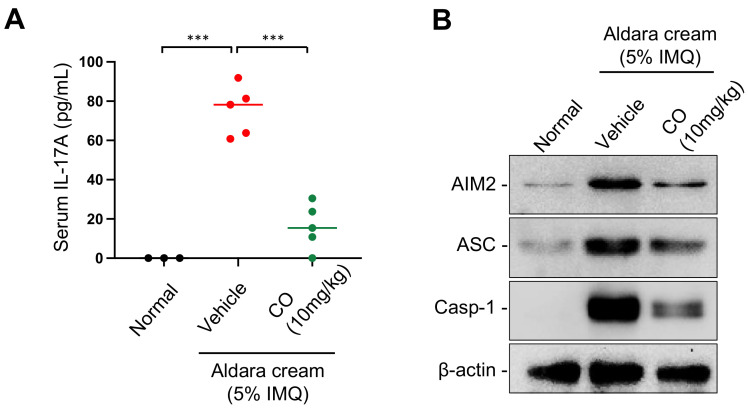
CO reduces IMQ-induced serum IL-17A elevation and the expression of AIM2 inflammasome components in skin tissue. CO and Aldara cream were applied as described in Figure 6C to obtain serum and prepare skin tissue extracts. (**A**) Serum IL-17A levels measured by an ELISA assay. Data represent the means ± SDs; *** *p* < 0.001. (**B**) Western blot analysis for the levels of AIM2 inflammasome components in skin extracts.

The data revealed that IMQ treatment caused an increase in IL-17A levels, which were significantly diminished by CO treatment (Figure 7A).

Moreover, as expected, we observed increased AIM2, ASC, and caspase-1 in IMQ- induced skin lesions, and CO treatment attenuated this increase in expression (Figure 7B).

These findings suggest that the reduction in skin inflammation by CO treatment may be due, at least in part, to the inhibition of IL-17A cytokine release and the suppression of AIM2 inflammasome activation.

## 3. Discussion

Inflammasomes are part of the innate immune system and are responsible for sensing danger signals from pathogens in damaged cells, which is essential for protecting the host from environmental threats [31]. However, dysregulated inflammasome activation has been implicated in the pathogenesis of various inflammatory diseases [46].

Therefore, many efforts have been made to explore inhibitors of inflammasome activation from various sources, including medicinal plants that have been traditionally used for their anti-inflammatory properties [47,48,49]. This study provides evidence for the inhibitory activity of CO, an ethanolic extract of *Cornus officinalis* seed, on AIM2-inflammasome activation and its potential therapeutic effect on psoriatic-like skin inflammation induced by IMQ, to which the AIM2 inflammasome is known to contribute [38,40].

The AIM2 inflammasome is responsible for sensing cytosolic dsDNA derived from both pathogens and damaged cells [15,50], and its activation participates not only in host protection but also in some inflammatory diseases, such as psoriasis, chronic kidney injury, and atherosclerosis [18,21,51].

To explore the underlying mechanisms that mediated the inhibitory effect of CO on AIM2-inflammasome activation, we investigated the impact of CO treatment on the series of steps required for inflammasome activation. Caspase-1 is a key enzyme that cleaves to the proinflammatory cytokines IL-1β and IL-18 in their active form, which contribute to triggering an inflammatory response [6]. Caspase-1 activation occurs within the inflammasome complex, where ASC speck is crucial for recruiting and activating caspase-1 [2,3,5]. Therefore, most inflammasome inhibitors show inhibitory activity towards ASC speck formation and subsequent caspase-1 activation [52,53]. The treatment of CO also exhibited the inhibition of both ASC speck formation and caspase-1 cleavage, indicating that its ability to inhibit the release of IL-1β from cells is attributable to the inhibition of inflammasome complex formation. Furthermore, CO also inhibited the formation of dsDNA-induced AIM2 speck in AIM2-overexpressing HEK293T cells, which is an essential step in the activation of the AIM2 inflammasome [33]. This observation is crucial in explaining why CO specifically inhibits the AIM2 inflammasome activation, since ASC speck formation and caspase-1 cleavage are also commonly observed in other inflammasome activation occurrences, such as NLRP1, NLRP3, and NLRC4 [4,6].

Some studies suggest that the AIM2 inflammasome activation is contributed to by the progression of psoriasis by the release of pro-inflammatory cytokines [38,40]. Psoriasis is a form of chronic, immune-mediated inflammatory dermatosis that affects approximately 2–4% of the population worldwide [54]. Despite the various therapies available, such as psoralen, steroids, ultraviolet (PUVA) photochemotherapy, immunosuppressants, and biological therapies, most treatments have various side effects and require long-term administration [55,56,57,58,59,60]. Therefore, the discovery of new substances that can treat psoriasis is an area of great interest. To explore the in vivo relevance of the inhibitory effect of CO on AIM2 inflammasome activation in cells, we investigated the effect of CO on skin inflammation in an imiquimod-induced psoriasis mouse model. In this study, psoriasis-like skin inflammation was induced by repeated topical application of Aldara cream containing 5% imiquimod [39], and treated mice exhibited typical psoriasis phenotypes, such as red colored plaques (erythema), silvery white dry scales, thickened skin, ear swelling, and bodyweight loss [61]. However, these psoriasis-like symptoms were alleviated by the topical application of CO. Histological analysis revealed that, as expected, CO ameliorates histological changes associated with imiquimod-induced psoriasis, such as parakeratosis, hyperpigmentation, and hair follicle destruction, which demonstrates its protective effect on imiquimod-induced psoriasis-like skin inflammation.

Furthermore, we found that CO application significantly reduced the expression levels of AIM2 inflammasome components, including AIM2, ASC, and caspase-1, in skin lesions, as well as serum IL-17A levels in IMQ-treated mice.

In previous studies, AIM2 has been shown to be upregulated in psoriatic skin lesions [21,40], and the blocking activation of the AIM2 inflammasome reduces inflammation and improves symptoms in animal models of psoriasis [62], suggesting that it may play a role in the chronic inflammation associated with the disease. Serum levels of IL-17A are increased, which increases are implicated in the pathogenesis of psoriasis [41,42,43,44,45].

The exact mechanism by which CO alleviates IMQ-induced skin inflammation is currently unknown, and the identification of active compounds with therapeutic efficacy and further research will be required in order to fully understand it. However, at least in part, the decrease in AIM2 inflammasome activation and IL-17A levels in serum may contribute to this effect.

Altogether, these results indicate that CO holds potential not only as a therapeutic material for the treatment of psoriasis and other diseases associated with the AIM2 inflammasome and cellular damage, but also as a source for further exploration of the effective active compound.

## 4. Materials and Methods

### 4.1. Reagents

The following reagents and materials were purchased for the study: LPS (L2630), from Pierce Chemical (Rockford, IL, USA); nigericin (tlrl-nig), nano-SiO2 (tlrl-sol), poly(deoxyadenylic-deoxythymidylic) acid (poly(dA:dT), tlrl-patn), Pam3CSK4 (tlrl-pm2s-1), lipofectamine 2000 (11668019), YVAD-CMK (Inh-yvad), and a mouse IL-1 beta ELISA kit (88-7013-88), from Invivogen (San Diego, CA, USA); a human IL-1 beta ELISA kit (DY201), from R&D systems (Minneapolis, MN, USA); flagellin (AG-40B-0095), from Adipogen International (San Diego, CA, USA); ATP (A7699), thiazolyl blue tetrazolium bromide (M5655), DMSO (D2660), KCl (P5405), and glycine (G7126), from Sigma-Aldrich (St. Louis, MO, USA); disuccinimidyl suberate (DSS, #21555) and a BCA assay kit (#23225), from Thermo Fisher Scientific (Walthan, MA, USA); RIPA lysis buffer (#89900), from iNtRON (Seoul, Republic of Korea); protease inhibitor (P3100-005), from GenDEPOT (Katy, TX, USA); RPMI1640 (11875119), DMEM (11995073), Opti-MEM (31985088), fetal bovine serum (16000-044), antibiotic-antimitotic (15240062), and trypsin/EDTA (15400-054), from Gibco (Grand Island, NY, USA); TNF-alpha (315-01A) and human interferon gamma (300-02), from PeproTech (Rocky Hil, NJ, USA); antibodies against ASC (AL177) and caspase-1 (p20) (AG-20B-0042), from Adipogen International (San Diego, CA, USA); antibodies against mouse IL-1 beta/IL-1F2 (AF401-NA) and NLRP3 (Cryo2), from R&D systems (Minneapolis, MN, USA); antibodies against β-actin (sc-1616), from Santa Cruz Biotechnology (Santa Cruz, CA, USA); antibodies against AIM2 (63660S) and GAPDH (sc-365062), from Cell Signaling Technology (Beverly, MA, USA); antibodies against FLAG (F1804), from Sigma-Aldrich (St. Louise, MO, USA); an LDH assay kit (DG-LDH1000) and a Western blot chemiluminescence reagent kit (EZ-Western Lumi Pico Kit, DG-WP250), from DoGenBio (Seoul, Republic of Korea); Nitrocellulose membranes (HATE00010), from Millipore Corporation (Bedford, MA, USA); and an ELISA MAX™ Deluxe Set Mouse IL-17A kit (432504), from BioLegend (San Diego, CA, USA).

### 4.2. Animals

Male BALB/c and C57BL/6 mice, six weeks old, were obtained from Orient Bio Co. (Seoul, Republic of Korea). The mice were housed in groups of five in a controlled environment (22 ± 2 °C, 55 ± 5% humidity, 12-hour light/dark cycle) with unlimited access to food and water. The experiments were conducted in accordance with the guidelines set by the Konkuk University Animal Care Committee and were reviewed and approved by the Ethics Committee of Konkuk University (approval number: KU22192).

### 4.3. Plant Materials

For the screening, crude extracts were obtained from the plant extract bank at the Republic of Korea Research Institute of Bioscience and Biotechnology (KRIBB) (Daejeon, Republic of Korea). The powder was then dissolved in dimethyl sulfoxide (DMSO) and diluted with cell culture media immediately before use. The final concentration of DMSO in the cell culture media was maintained below 0.1%.

The preparation of samples for follow-up studies was as follows. The seeds of *Cornus officinalis* were ground and extracted with 100% ethanol at room temperature for 48 h and filtered through a filter paper (Whatman ND, No. 41, Grade 5). After evaporation, the extracts were dissolved in distilled water and lyophilized.

### 4.4. General Experimental

The ^1^H NMR spectrum was recorded at 300 MHz in DMSO-*d*_6_ on Varian Inova spectrometers. The ^13^C NMR spectrum was obtained at 100 MHz in DMSO-*d*_6_ using an Agilent NMR spectrometer. Low-resolution LC-MS data were acquired using an Agilent Technologies 1260 quadrupole LC/MS system, equipped with a diode array detector (DAD) and a Phenomenex Luna C18 (2) 100 Å, 50 mm × 4.6 mm, 5 μm, at a flow rate of 1.0 mL/min, at the National Research Facilities and Equipment Center (NanoBioEnergy Materials Center) at Ewha Womans University. The fractions were purified by reversed-phase high-performance liquid chromatography (HPLC) (Phenomenex Luna C18 (2), 100 Å, 250 nm × 10 mm, 5 μm).

### 4.5. LC-MS Fingerprinting Analysis and Isolation of Compound

The dried powder of 10 mg CO was added to a 1.5 mL Eppendorf tube. Subsequently, 1 mL of water was added and mixed. The sample was filtered through a 0.2 μm filter and injected into an LC-MS system for analysis. A binary gradient elution system composed of 1% trifluoroacetic acid in water and acetonitrile was employed. The LC-MS data were acquired using the following gradient: 0–10.0 min, 13% acetonitrile; 10.0–40.0 min, 13–15% acetonitrile; 40.1–45.0 min, 100% acetonitrile; 45.1–48.0 min, 5% acetonitrile. The following chromatogram and content assays for CO were performed using the above liquid-phase conditions.

The CO (2 g) was subjected to open column chromatography purification in an RP C18 flash column by step gradient elution of methanol/H_2_O from 20% to 100% of methanol, subsequently, to afford 8 fractions (SSU-F1 ~ SSU-F8). Fraction SSU-F3 (22.5 mg) (H_2_O: MeOH = 50:50) was purified by reversed-phase HPLC (Phenomenex Luna C-18 (2), 250 × 100 mm, 2.0 mL/min, 5 μm, 100 Å, UV = 254 nm) using an isocratic solvent system with 17% acetonitrile in water to yield ellagic acid (1 mg) as white powder. Its molecular formula was deduced based on the LC-MS data and 1H NMR data.

Ellagic acid: white powder; 1H NMR (300 MHz, in DMSO-d6) δH 7.46 (2H, s); 13C NMR (100 MHz, in DMSO-d6) δC 159.1, 148.1, 139.5, 136.4, 112.3, 110.3, 107.7; LR-ESI-MS m/z 303.07 [M + H]+.

### 4.6. Cell Culture

HEK293T, HaCaT, and L929 cells were cultivated in DMEM medium supplemented with L-glutamine, 10% FBS, and 100 U/mL antibiotic–antimitotic. For L929 conditioned medium (LCM), 1.3 × 10^6^ cells were cultured for 7 days in a T175 cell culture flask, and the culture supernatants were filtered through 0.22 μm and kept at −80 °C until needed.

Bone marrow cells were prepared from femurs, and red blood cells were removed by incubation with RBC lysis buffer (150 mM NH_4_Cl, 10 mM KHCO_3_, 120 μM monosodium EDTA, pH 7.3). The bone marrow cells were suspended in complete medium (RPMI 1640 supplemented with 10% FBS, 1% penicillin/streptomycin, 50 μM 2-mercaptoethanol, 1 mM sodium pyruvate, MEM-NEAA, 30% LCM medium). The cells were cultured in a 150 cm^2^ dish at a density of 5 × 10^6^ cells/20 mL, and at days 3 and 6 the medium was replaced with fresh medium. At day 7, the macrophages were detached and seeded for further experiments.

### 4.7. Cytotoxicity Assay

Cells were seeded in 96-well plates and cultured overnight. Then, the cells were incubated with the indicated concentrations of CO for 24 h. For the MTT assay, the culture medium was discarded and replaced with MTT solution (500 μg/mL) in culture medium, followed by 2 h incubation at 37 °C. Then, DMSO was added to solubilize the formazan, and absorbance (OD) was measured at 550 nm using a Multiskan GO Microplate spectrophotometer (Thermo Fisher Scientific, Waltham, MA, USA). The viability was calculated by relative comparison with control cells. The cell viability was calculated as % = (OD of each sample/OD of non-treated control) × 100.

### 4.8. Inflammasome Activation in BMDMs

The BMDMs were pre-treated with either LPS (100 ng/mL) or Pam3CSK4 (300 ng/mL) for 3 h. After priming, the medium was replaced with Opti-MEM, and the cells were incubated with or without various samples before stimulation with various inducers: ATP (5 mM) or nigericin (10 μM) for 1 h, silica (150 μg/mL) for 3 h, transfection with poly(dA:dT) (1 μg/mL) for 2 h, and flagellin (1 μg/mL) or LPS (50 μg/mL) for 3 h using lipofectamine 2000.

### 4.9. Immunoblot Analysis

The amounts of protein in cell lysates were determined using a BCA assay kit; cell lysates and cell supernatants were boiled for 5 min with sample buffer. Equivalent amounts of samples from cell lysates and cell supernatants were separated by SDS-PAGE and transferred onto nitrocellulose membranes. Blocked membranes were incubated with primary antibodies overnight, and the membranes were incubated with horseradish peroxidase (HRP)-conjugated antibody for 1 h at room temperature. After incubation with secondary antibodies, the membranes were developed for visualization using an Enhanced chemiluminescence (ECL) detection kit by Image Analyzer (Bio-Rad, Hercules, CA, USA, Clarity Western ECL substrate, #1705061).

### 4.10. Enzyme-Linked Immunosorbent Assay (ELISA)

Cell supernatants were collected for measuring IL-1β levels using an ELISA kit according to the manufacturer’s instructions. Mouse sera were collected for measuring IL-17A levels using an ELISA kit, according to the manufacturer’s instructions. The absorbance (OD) was measured at 450 nm using a Multiskan GO Microplate spectrophotometer (Thermo Fisher Scientific, Waltham, MA, USA).

### 4.11. Lactate Dehydrogenase (LDH) Assay

Cell death was determined by measuring LDH release into culture supernatants using an EZ-LDH kit (DoGen Bio, Seoul, Republic of Korea), according to the manufacturer’s instructions. The absorbance (OD) was measured at 450 nm using a Multiskan GO Microplate spectrophotometer (Thermo Fisher Scientific, Waltham, MA, USA). The LDH release was calculated as % = (OD of each sample/OD of lysis control) × 100.

### 4.12. Separation of Cell Lysates into Soluble and Insoluble Fractions of Triton X-100

Cells were lysed with TTNE lysis buffer (1% Triton X-100, 150 mM NaCl, 50 mM Tris, and 2 mM EDTA and containing a protease inhibitor cocktail) on ice for 20 min and centrifuged at 13,000 rpm for 15 min. The supernatants (soluble fractions) and pellets (insoluble fractions) were extracted using 1% SDS lysis buffer (20 mM Tris (pH 7.5), 150 mM NaCl, 1% SDS).

### 4.13. ASC Oligomerization Assay

For the ASC oligomerization assay, cells were lysed in TTNE buffer (1% Triton X-100, 50 mM Tris-HCl, pH 7.4, 150 mM NaCl, 2 mM EDTA, and complete protease and phosphatase inhibitor cocktail) on ice for 20 min. The cell lysates were centrifuged at 6000 rpm at 4 °C for 15 min. Pellets were washed with and resuspended in PBS and cross-linked using 2 mM disuccinimidyl suberate (DSS) at room temperature for 30 min, and were then denatured in 20 mM Tris-HCl (pH 7.4) for 15 min at room temperature. The cross-linked pellets were centrifuged at 13,000 rpm for 15 min and dissolved in SDS sample buffer.

### 4.14. Immunofluorescence Staining for ASC Specks

BMDMs seeded on four-well chamber slides were primed and pre-treated for 30 min with or without inhibitors before transfection with poly(dA:dT) (1 μg/mL) for 2 h. The slides were transferred to 4% paraformaldehyde for 20 min on ice and permeabilized with 100% acetone for 10 min at −20 °C. The dried slides were rehydrated with PBS and blocked with 10% horse serum for 1 h. The cells were stained with anti-ASC antibodies (AL177, Adipogen, San Diego, CA, USA) and Cy3-conjugated anti-rabbit antibodies. Nuclei were stained with DAPI, and fluorescence microscopy (AX10, Zeiss, Oberkochen, Germany) images were obtained.

### 4.15. Immunofluorescence Staining of AIM2 Specks

HEK293T cells cultured on poly-L-Lysine-coated cover glass were transfected with pCMV2-FLAG-mAIM2 plasmids (Addgene_51450) using lipofectamine 2000 for 24 h. Moreover, the cells were pre-treated with CO (10 μg/mL) for 30 min, followed by transfection with poly(dA:dT) (1 μg/mL) for 4 h. The slides were transferred to 4% paraformaldehyde for 20 min on ice and permeabilized with 100% acetone for 10 min at −20 °C. Then, the dried slides were rehydrated with PBS and blocked with 10% horse serum for 1 h. The cells were stained with anti-FLAG antibodies (F1804, Sigma-Aldrich) and Cy3-conjugated anti-rabbit antibodies. Nuclei were stained with DAPI, and fluorescence microscopy (AX10, Zeiss, Oberkochen, Germany) images were obtained.

### 4.16. Inflammasome Activation in HaCaTs

The HaCaTs were pre-treated with IFN-γ (100 ng/mL) and TNF-α (10 U/mL) for 24 h. The primed cells were incubated with or without CO or YVAD for 30 min and stimulated with poly(dA:dT) (1 μg/mL) for 24 h.

### 4.17. IMQ-Induced Mouse Psoriasis

Psoriasis-like dermatitis was induced in mice by topical administration of Aldara cream (50 mg/day) on a 2 cm × 3 cm area of the back and 10 mg/day on the right ear for 8 consecutive days. CO mixed in Vaseline cream was topically applied 4 h before the Aldara cream application.

The study animals were divided into four experimental groups: group 1: control group (*n* = 4), mice received Vaseline cream without Aldara cream application; group 2: vehicle group (*n* = 4), mice received Vaseline cream with Aldara cream application; group 3: CO 5 mg/kg (*n* = 4), mice received CO (5 mg/kg) in Vaseline cream with Aldara cream application; group 4: CO 10 mg/kg (*n* = 4), mice received CO (10 mg/kg) in Vaseline cream with Aldara cream application. The animals were monitored for the severity of their skin conditions over 8 consecutive days according to the Psoriasis Area and Severity Index (PASI) with a score range of 0–4 (0, none; 1, mild; 2, moderate; 3, severe; 4, very severe) for erythema and scaling. In addition, the thickness of skinfolds on the back and ear were also measured using a caliper (accuracy: ±0.02 mm; Mitsutomo, Tokyo, Japan).

On the eighth day, the animals were sacrificed, their back skins were excised, and the biopsies of lesional skin were fixed in formalin and embedded in paraffin. The paraffin-embedded biopsies were sectioned and stained with hematoxylin and eosin (H&E) for a histological analysis.

### 4.18. Statistical Analysis

Data are expressed as means ± standard errors of the means (SEMs) or as the standard deviations (SDs) of at least three independent experiments, each performed in triplicate. Statistical analysis was based on Dunnett’s post hoc test via Graph Pad PRISM 5.0 software (San Diego, CA, USA), and *p*-values less than 0.05 were considered to indicate statistically significant differences.

## Data Availability

Not applicable.

## Supplementary Materials

The following supporting information can be downloaded at: https://www.mdpi.com/article/10.3390/ijms24065653/s1.
