# Supplementary material for: Cornus officinalis Seed Extract Inhibits AIM2-Inflammasome Activation and Attenuates Imiquimod-Induced Psoriasis-like Skin Inflammation
Source: Int J Mol Sci. 2023 Mar 15;24(6):5653. doi: 10.3390/ijms24065653 (PMC10051512; doi:10.3390/ijms24065653)
Supplement: Supplementary file 1 [file ijms-24-05653-s001.zip › ijms-2259886-supplementary.pdf]

Supplementary Materials

Cornus officinalis Seed Extract Inhibits AIM2-Inflammasome Activation and Attenuates Imiquimod-Induced Psoriasis-like Skin Inflammation

Se-Bin Lee [1], Ju-Hui Kang [1], Eun-Jung Sim [1], Ye-Rin Jung [1], Jeong-Hyeon Kim [2], Prima F. Hillman [2], Sang-Jip Nam [2] and Tae-Bong Kang [1,3,*]

[1] BK21 Project Team, Department of Applied Life Science, Graduate School, Konkuk University, Chungju 27478, Republic of Korea
[2] Department of Chemistry and Nanoscience, Ewha Womans University, Seoul 03760, Republic of Korea
[3] Department of Biotechnology, Research Institute of Inflammatory Diseases, Research Institute (RIBHS), College of Biomedical and Health Science, Konkuk University, Chungju 27478, Republic of Korea
* Correspondence: kangtbko@kku.ac.kr; Tel.: +82-043-840-3904

Table of Contents

Figure S1. LC-MS spectrum of CO. S3

Figure S2. ^{1}H NMR spectrum (300 MHz) of ellagic acid in DMSO-d_6. .... S4

Figure S3. ^{13}C NMR spectrum (100 MHz) of ellagic acid in DMSO-d_6. .... S5

Figure S4. LC chromatographs of CO and standard chemical of ellagic acid in 254 nm S6

Figure S5. LC chromatographs of CO and standard chemical of methyl gallate in 280 nm S7

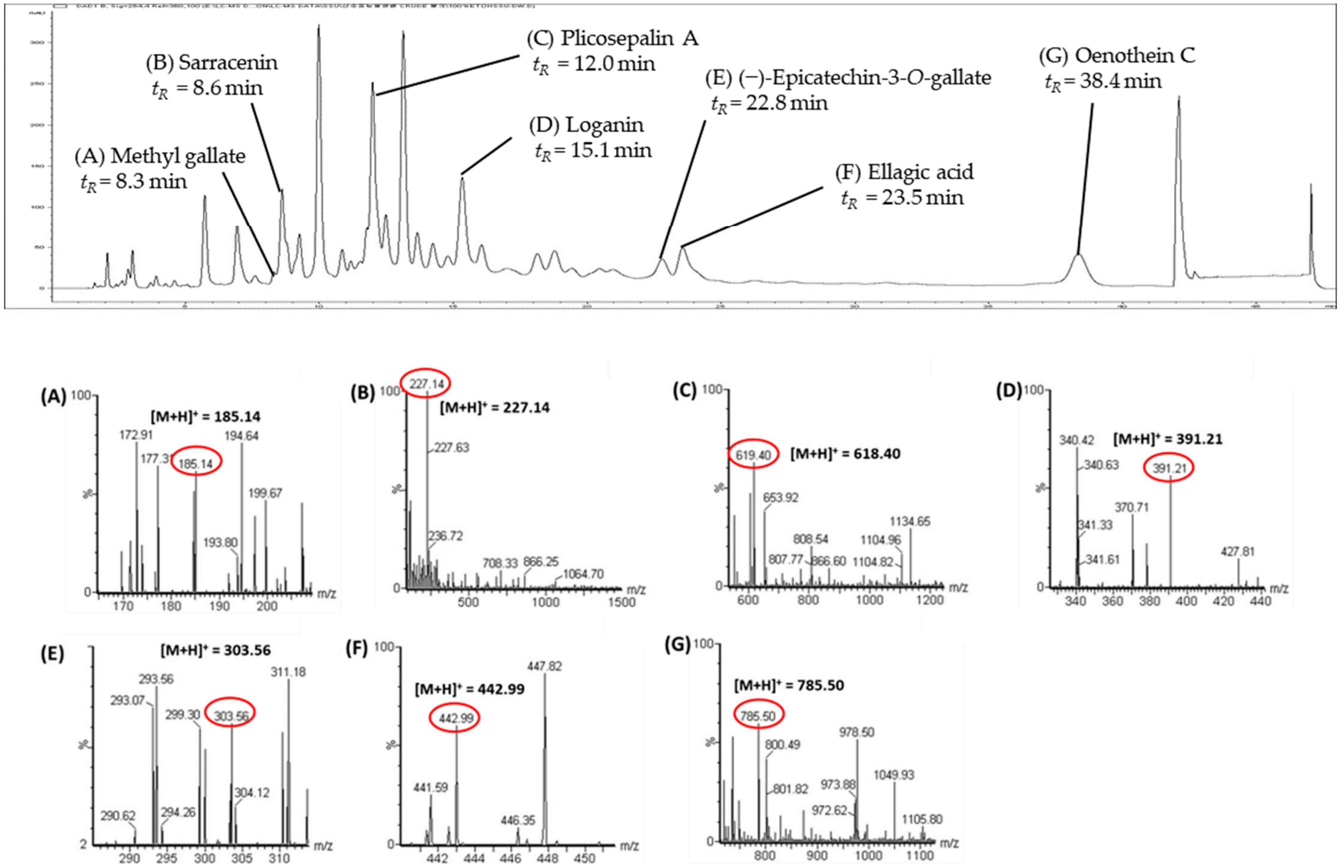

Figure S1. LC-MS spectrum of CO.

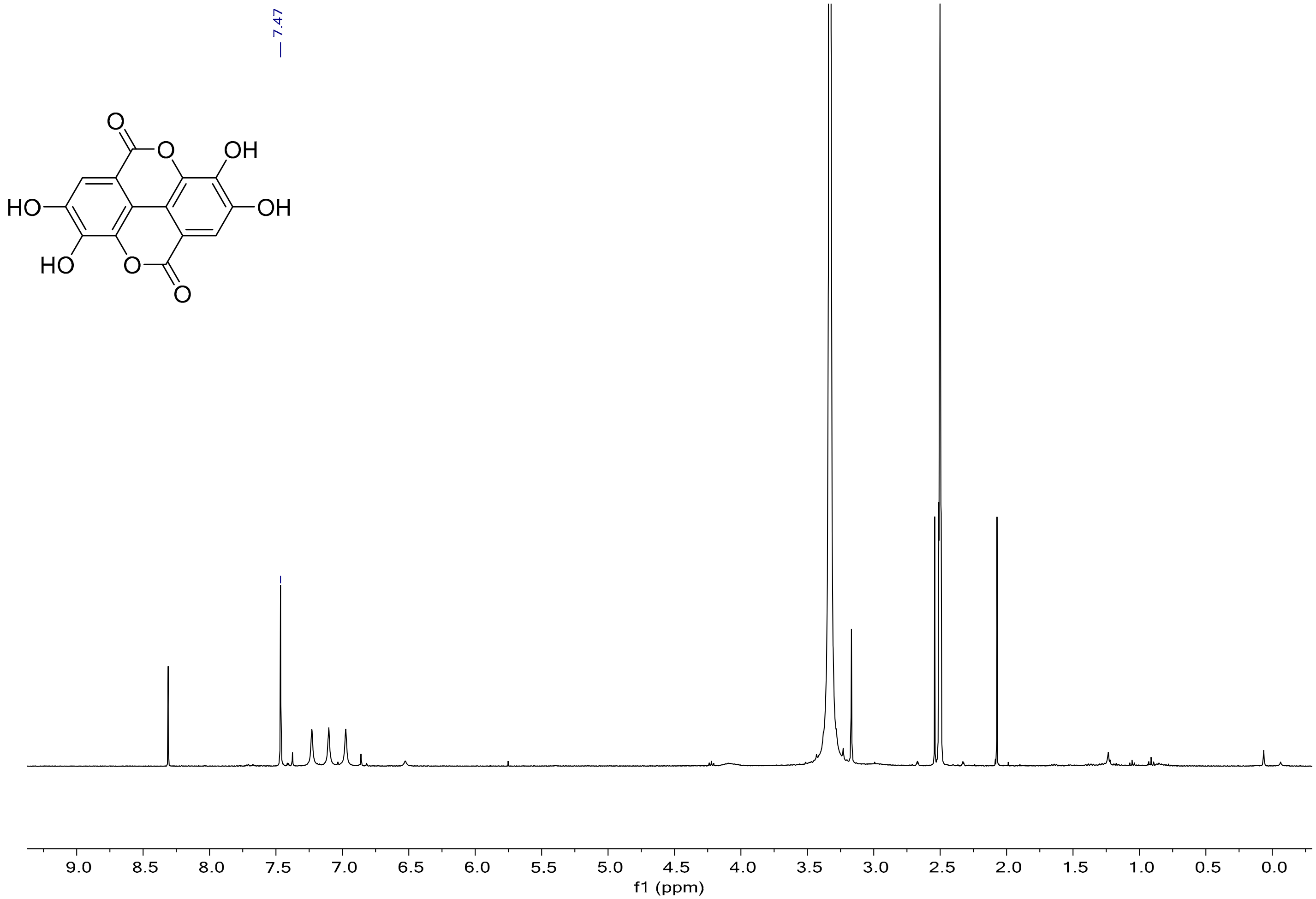

Figure S2. ^{1}H NMR spectrum (300 MHz) of ellagic acid in DMSO-d_6.

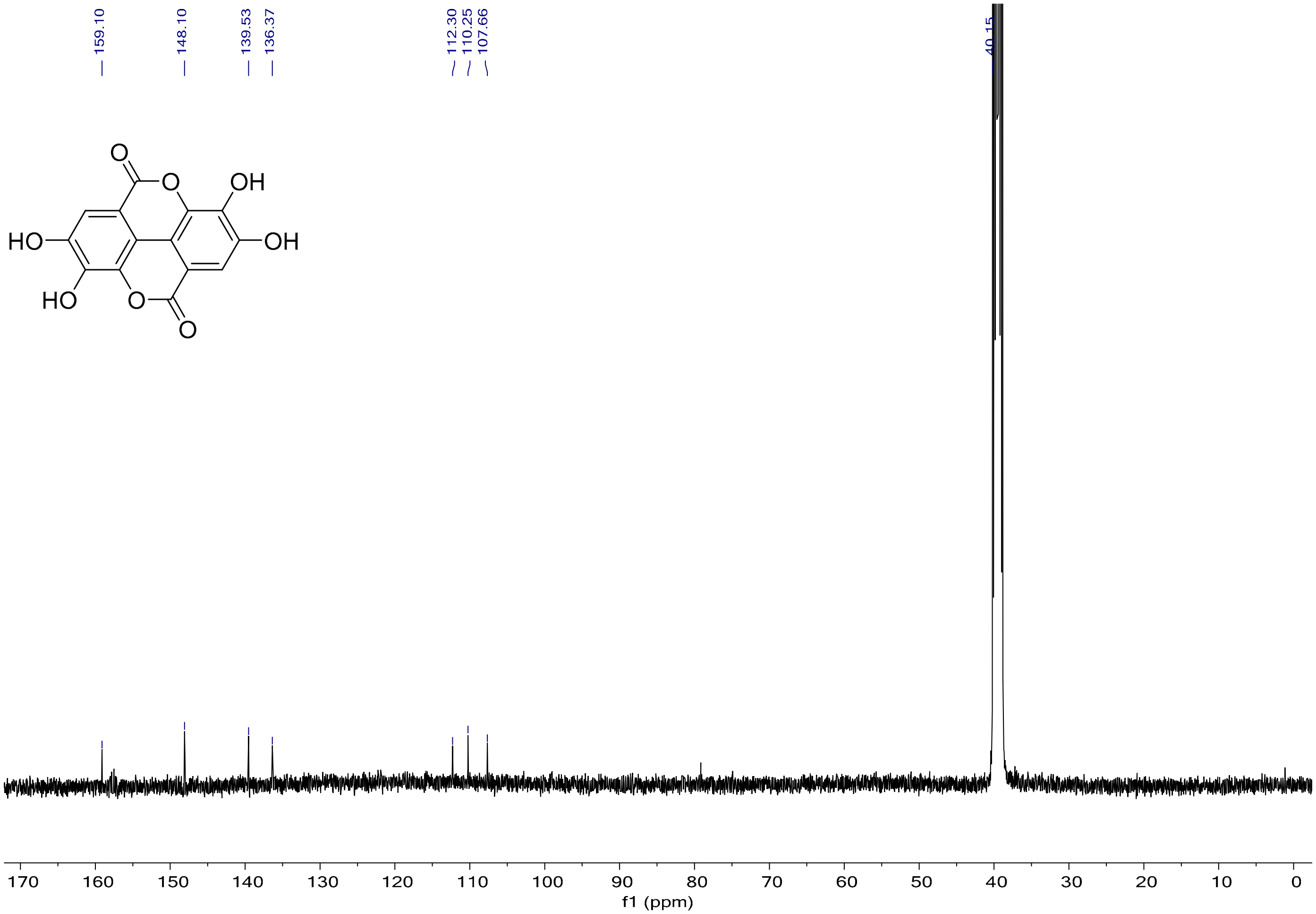

Figure S3. ^{13}C NMR spectrum (100 MHz) of ellagic acid in DMSO-d_6.

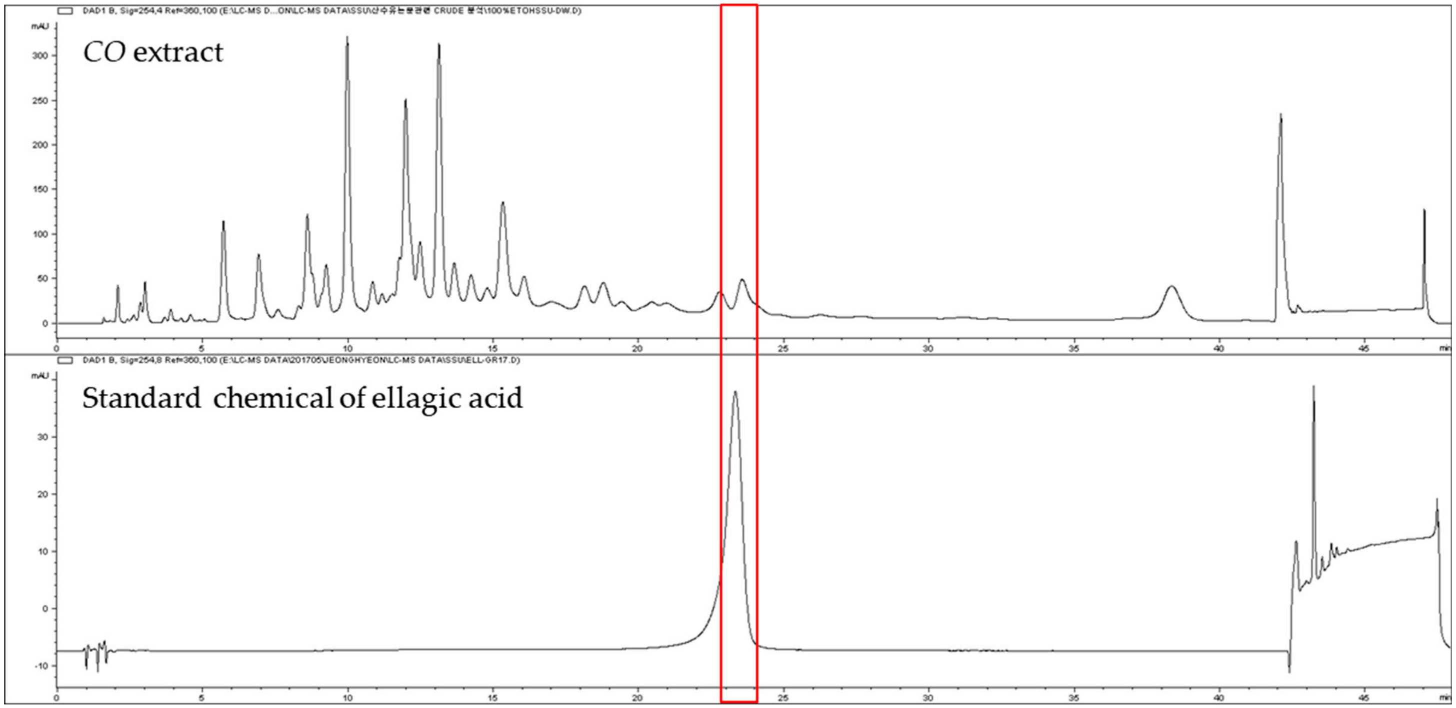

Figure S4. LC chromatographs of CO and standard chemical of ellagic acid in 254 nm.

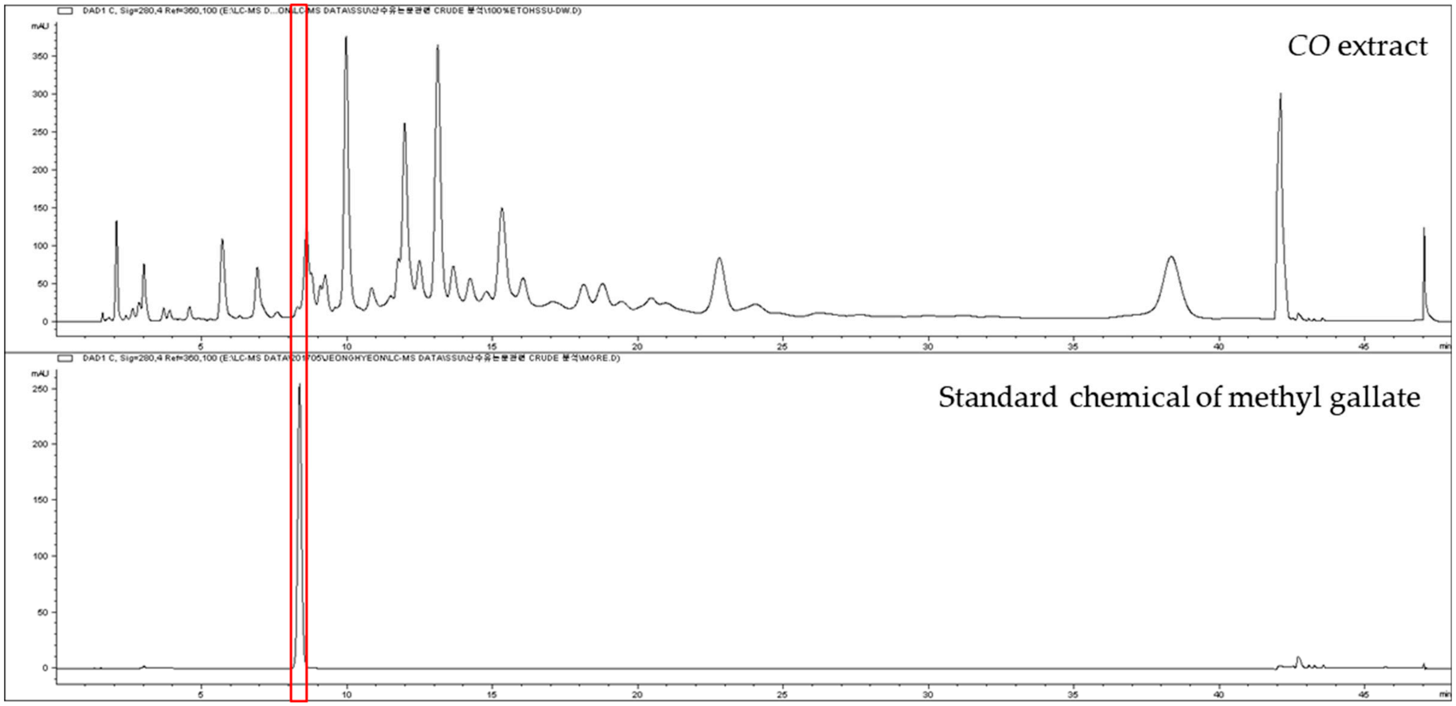

Figure S5. LC chromatographs of CO and standard chemical of methyl gallate in 280 nm.
